# 2D mesoporous materials

**DOI:** 10.1093/nsr/nwab108

**Published:** 2021-06-22

**Authors:** Yan Ai, Wei Li, Dongyuan Zhao

**Affiliations:** Department of Chemistry, Laboratory of Advanced Materials, Shanghai Key Laboratory of Molecular Catalysis and Innovative Materials, and State Key Laboratory of Molecular Engineering of Polymers, Fudan University, China; Department of Chemistry, Laboratory of Advanced Materials, Shanghai Key Laboratory of Molecular Catalysis and Innovative Materials, and State Key Laboratory of Molecular Engineering of Polymers, Fudan University, China; Department of Chemistry, Laboratory of Advanced Materials, Shanghai Key Laboratory of Molecular Catalysis and Innovative Materials, and State Key Laboratory of Molecular Engineering of Polymers, Fudan University, China

## Abstract

2D mesoporous materials, combining the unique features of 2D and mesoporous structures, represent a new research direction in materials science. This perspective article highlights the latest progress of 2D mesoporous materials.

Mesoporous materials with high surface areas, large pore volumes, tunable nanostructures and diverse compositions have been widely investigated, and show great promise in adsorption, catalysis, separation, biomedicine, energy storage and conversion, especially in chemical processes involving large molecules [1]. Although great progress has been achieved, how to accurately design and synthesize mesoporous materials is still the main theme of current research, to better understand the structure–performance relationship and to meet the ever-growing demand with regard to diversified applications. Typically, mesoporous materials can be extended into various dimensions through the bottom-up self-assembly approach, but most of them are centered in spherical and bulk solids.

Since the first reported isolation of graphene, 2D materials have attracted considerable attention due to their fascinating optical, electrical and mechanical properties, which open up a new research area of unexplored fundamental science [2]. Typically, 2D materials are atomically thick sheets that theoretically possess high surface areas, which generally translate into high reactivity and quantum effect. However, it is easy for 2D materials to form a densely stacked structure, thus greatly impeding the mass transfer and the full utilization of their active surfaces.

2D mesoporous materials, combining the unique features of 2D and mesoporous structures, represent a new research direction in materials science. The introduction of ordered mesopores can not only effectively tune the electric structures, but also open up the in-plane transfer pathway and increase the accessible surface sites of 2D materials. On the other hand, pushing mesoporous materials into an extended lateral dimension forms unique 2D ultrathin nanosheets that can effectively address the limitations of bulk counterparts, such as the inaccessible surface areas, unsatisfactory mass transfer resistance, and so on [3]. 2D mesoporous materials, including intrinsically layered or non-layered nanosheets, have shown very fascinating properties and significantly improved performances in various application scenarios.

Many attempts have been made to synthesize 2D mesoporous materials through the top-down approach. In this case, the nanosheets are firstly constructed, then the in-plane mesopores are usually generated by post etching or the structural transformation method [4]. Although this approach generally leads to atomically thin nanosheets, they have disordered porous structures, with defects. Moreover, this way is complex, time-consuming and uncontrollable. Following the bottom-up synthesis of 2D materials, the most straightforward and feasible method of preparing 2D mesoporous materials is the micelle-guided self-assembly strategy (Fig. 1) [5]. The first key issue is to build an interface where the micelles can two-dimensionally self-assemble like classic chemical vapor deposition synthesis of graphene on metallic catalysts. The interface between two phases, typically including gas–liquid, liquid–liquid and solid–liquid, provides the infinite space for the 2D growth process (Fig. 1). The second vital issue is organizing micelles and framework building blocks into a periodical arrangement along the lateral dimension without growth in the vertical direction. The third challenge is the controllable polymerization and crystallization of the pore wall without interfering with the assembly process. It is worth noting that this approach is very simple and reproducible, and has recently attracted considerable attention for 2D mesoporous materials.

**Figure 1.**
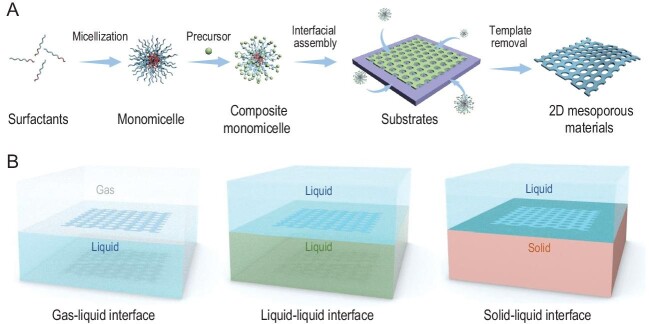
Schematic illustration of the (A) micelle-guided self-assembly strategy for 2D mesoporous materials and (B) the typical interfaces for 2D self-assembly, including gas–liquid, liquid–liquid and solid–liquid interfaces.

For example, Fang *et al.* first reported the synthesis of 2D mesoporous carbon materials through a solution deposition method [6]. Composite monomicelles are first constructed as the building block. Anodic aluminum oxide (AAO) membranes are explored to provide a solid interface for the monomicelles close-packing assembly in solution. The 2D mesoporous carbon nanosheets can be obtained after carbonization and etching away of AAO; they have ultrathin thickness, high surface areas and ordered mesopores, and show very good performance for lithium-ion batteries. This study initiated the research of 2D mesoporous materials. Later, 2D mesoporous TiO_2_ nanosheets were synthesized in a mixture solvent (ethanol and glycerol) [7]. The liquid–liquid interfacial confinement effect of highly adhered glycerol enables the oriented assembly of monomicelles into a 2D mesostructure with controllable thickness. More recently, Wiesner and coworkers built a clear interface between two immiscible solvents for fabrication of 2D mesoporous silica superstructures [8]. The uniform silica nanocage subunits, and large and stable liquid−liquid interfacial area create the ability to precisely control structure and composition in the 2D silica nanosheets. This study provides new insights into how to manipulate the micelles self-assembly in two dimensions.

In addition to liquid–liquid and solid–liquid interfaces, gas–liquid interfaces have also been explored to synthesize 2D mesoporous materials. For instance, well-defined 2D mesoporous Ir nanosheets have been synthesized through a gas-directing close-packed assembly of monomicelles [9], in which CO gas can bind strongly to the basal facets of Ir, forming a microscale interface and thus enabling the metal nanosheets to grow in a 2D manner. The resulting 2D Ir nanosheets are polycrystalline and riddled with abundant mesopores, and more importantly highlight the unique catalytic behavior of 2D mesoporous materials by maximizing exposed surface atoms. In a more recent example, a polymer–polymer interfacial self-assembly strategy has been developed to prepare 2D mesoporous carbon nanosheets without additional substrates [10]. Starting from a homogeneous solution, the block copolymer-directed self-assembly is confined on the interface between two immiscible homo-polymer major phases with ultrathin thickness to decrease the interfacial tension with solvent evaporation. This new approach is simple and straightforward, and opens a window for controllable synthesis.

2D mesoporous materials are still in the infancy stage. Convenient and universal synthetic methods as well as a deep understanding of forming mechanisms urgently need to be explored for precise synthesis of 2D mesoporous materials with controllable compositions, geometries and pore sizes. It can be predicted that this area would be greatly expanded by the development of ordered mesoporous atomically thin sheets, such as graphene, hexagonal boron nitride, transition metal dichalcogenides, MXene, graphitic carbon nitride and beyond. We believe that this dream will come true in the near future. Another enticing possibility is that, like the well investigated 2D heterostructures and composites, 2D mesoporous heterostructures and composites would open up a new hitherto unavailable design space for materials science. A breakthrough in the application of 2D mesoporous materials is highly expected, such as in condensed-matter physics, membrane separation, catalysis, energy storage, solar cells and electronic devices, but the fabrication of 2D mesoporous materials needs to be scale up in the future.
